# What works to prevent violence against children in Afghanistan? Findings of an interrupted time series evaluation of a school-based peace education and community social norms change intervention in Afghanistan

**DOI:** 10.1371/journal.pone.0220614

**Published:** 2019-08-06

**Authors:** Julienne Corboz, Wahid Siddiq, Osman Hemat, Esnat D. Chirwa, Rachel Jewkes

**Affiliations:** 1 Independent Research Consultant, Kabul, Afghanistan; 2 Help the Afghan Children, Kabul, Afghanistan; 3 School of Public Health, University of Witwatersrand, Johannesburg, South Africa; 4 Gender and Health Research Unit, South African Medical Research Council, Pretoria, South Africa; The University of New England, AUSTRALIA

## Abstract

**Background:**

Against a backdrop of more than four decades of war, conflict and insecurity, Afghanistan is recognised as suffering from endemic violence and children are exposed to multiple forms of violence, including at the family and school levels. This paper presents the results of an evaluation of school-based peace education and a community-based intervention to change harmful social norms and practices related to gender and the use of violence in conflict resolution, implemented in Afghanistan with the aim of reducing violence against and between children.

**Methods:**

The evaluation consisted of a cross-sectional, interrupted time series design with three data collection points over 12 months. Data was collected from students in 11 secondary schools (seven girls’ and four boys’ schools) in Jawzjan province of Afghanistan, with a total of 361 boys and 373 girls sampled at endline. All children were interviewed with a questionnaire developed for the study. Key outcomes included children’s experience of peer violence (both perpetration and victimization) at school, corporal punishment both at home and at school, and observation of family violence. Other outcomes included children’s gender equitable attitudes, attitudes towards child punishment, depression and school performance.

**Results:**

Between baseline and endline evaluation points, there were significant reductions in various forms of violence at the school level, including both boys’ and girls’ past month experience of peer violence victimization, peer violence perpetration, and corporal punishment by teachers. There were also significant reductions in boys’ and girls’ experience of corporal punishment at home and observation of family violence, with a particularly strong effect observed among girls. Both boys and girls had significantly more equitable gender attitudes and significantly less violence-supportive attitudes in relation to children’s punishment, and significantly fewer symptoms of depression. Girls’ school attendance was also significantly higher at endline.

**Discussion:**

To our knowledge this is the first time that a peace education program has been evaluated in Afghanistan, with or without a community intervention to change harmful social norms and practices related to gender and the use of violence for conflict resolution. The evaluation suggests that the intervention may have led to a reduction in various forms of violence, including children’s peer violence, corporal punishment of children both at school and at home, and in children’s reports of domestic violence against women at the household level.

## Introduction

Afghanistan has experienced more than four decades of conflict, leading to massive disruptions in the development of education, health, livelihoods and infrastructure, and the stability of political governance. Against a backdrop of war, conflict and insecurity, Afghan society is recognised as suffering from endemic violence and children are exposed to multiple forms of violence. According to a global study conducted by UNICEF [1], Afghanistan is one of the few countries in which children are more likely to be disciplined using violent physical methods (for example, kicking, slapping, spanking, beating or other forms of corporal punishment) rather than violent psychological methods (for example, verbal aggression, threats, intimidation or humiliation). Although globally, approximately 17% of children are subjected to extreme forms of corporal punishment, more than a third of children in Afghanistan are subjected to extreme violence, and 78% of children aged 5 to 14 were reported to have experienced any violent discipline (psychological or physical) in the past month [1]. Children are also exposed to domestic violence against women in their households. The Afghanistan Demographic and Health Survey [2] found that the national prevalence of intimate partner violence (physical, emotional or sexual violence) against married women aged 15 to 49 was 56%, ranging from between 7% and 92% depending on the provincial context.

Violence against children in Afghanistan occurs at many levels, including the family and school levels. At the family level, the Central Statistics Organization and UNICEF [3] found that 74.2% of children aged between 2 and 14 years of age had experienced at least one form of physical or psychological punishment in the home in the past month and 38.4% of children had experienced extreme physical punishment. The physical punishment of children as a form of discipline, including slapping, ear-pulling, kicking, punching, or beating with sticks or other objects, is accepted as a normal part of parent-child relationships, despite many parents acknowledging the physical and psychological harm that such punishment can cause [4]. Smith [4] found that while boys overall are perceived to be physically punished more than girls due to boys being ‘naughtier’ than girls, mothers reported higher levels of violence against girls than fathers.

At the school level, children experience violence both at the hands of teachers and other students. Corporal punishment of students by teachers is highly prevalent, with one study by Save the Children [5] indicating that in 100% of boys’ schools and 20% of girls’ schools in the study sample, teachers were observed to use physical punishment against students. Furthermore, the majority of teachers reported believing that physical punishment was an important and inevitable form of maintaining discipline in the classroom. The same study found alarming rates of sexual abuse in boys’ schools, including rape by male teachers and sexual abuse of younger boys by older boys [5]. A more recent study by Save the Children also found that children reported male teachers and older students to be the main perpetrators of sexual abuse of boys in schools [6].

There has been very little research done on peer violence and bullying in Afghanistan; however, the available evidence suggests that violence perpetrated by children is also highly prevalent in schools. Data from the Global School-based Student Health Survey suggests that prevalence of past month bullying in Afghanistan is 44.2% [7]. A smaller study conducted in five provinces found that the prevalence of peer victimization was as high as 63%, although this included peer violence in different settings, with 18% of children reporting that they had been victimized by other children at school [6]. Although these figures relate to direct peer violence, violence enacted by children in schools may sometimes be mediated or institutionally supported by the school. For instance, Save the Children found that schools had disciplinary committees consisting of teachers and students who were fully authorised to physically punish students [5].

Violence against children occurs within a context of limited legal structures related to the protection of children. The Government of the Islamic Republic of Afghanistan (GoIRA) ratified the UN Child Rights Convention in 1994; however, progress has been slow due to a range of factors, including weak enforcement and rule of law, contradictions between some statutes of the convention and the Afghan constitution, and the application of provisions from customary and Sharia law in courts in the formal justice system [8]. Article 39 of the Education Act prohibits corporal punishment (including physical and psychological punishment) in schools [9]; however, other laws accept corporal punishment of children at home or at school. Article 54(1) of the Penal Code 1976 states that it is acceptable for fathers and teachers to punish sons and students provided the punishment is within the limits of religious and other laws (it does not make any explicit reference to girls or mothers), and article 194(6) of the Shiite Personal Status Law 2009 states that parents and legal guardians can discipline their children to the extent that compensation for injury is not required [9].

Peace education programs may be a useful way of reducing attitudes and practices that support and reproduce violence against and between children. Such programs have been implemented in many countries, both those not experiencing conflict and those in intractable conflicts, usually with the aim of fostering more peaceful, less violent, less intolerant and more just societies through supporting a positive change in values, attitudes and behaviours that reproduce violence and conflict [10, 11, 12]. Peace education programs can be implemented both at the school and broader community levels, although there has been a focus in recent decades on targeting children though school-based peace education curricula [13, 14]. This is due to a number of reasons, including targeting children as they are in their formative years and may be more open to change in attitudes and behaviours, and also because schools provide an institutional setting that has the authority and legitimacy for developing educational outcomes [11]. The literature suggests that few peace education interventions target schools and communities simultaneously, despite broader community and societal change likely requiring changes in beliefs, attitudes and behaviours beyond the boundaries of schools [15, 16].

A number of authors have described challenges to evaluating peace education, including a tendency for evaluators to test knowledge obtained rather than a change in violent attitudes and behaviours, and a lack of methods that allow for an understanding of how peace education can lead to change at the broader societal level [10, 12, 14]. Some studies have shown that peace education has a positive impact on children’s attitudes and beliefs, including more equitable and tolerant attitudes; however, very few evaluations have measured behaviour change and so it unknown if children’s attitudes lead to less violent practices and more peaceful conflict resolution [14, 17]. Some authors have also raised the challenge of lack of longitudinal data to understand whether the benefits of peace education are durable over time [12]. Rosen and Solomon [18] found that in the face of the intractable Palestinian/Israeli conflict, peace education was able to influence children’s peripheral beliefs over time, but not their core beliefs related to narratives of conflict.

In order to prevent violence perpetrated against children and between children and to lay the foundations for building peace across the society, Help the Afghan Children (HTAC) has been implementing peace education programming in schools and communities in Afghanistan since 2003. Peace education has been complemented by community interventions aimed to promote more equitable gender norms and reduce the use of violence against women and children. Although HTAC’s program outcomes have not been formally published, anecdotal data and monitoring and evaluation (M&E) data have indicated large reductions (up to 93%) in student’s aggressive behaviour (i.e. fighting, bullying and harassment) and large increases in the proportion of teachers abandoning corporal punishment [19, 20]. More recently, the What Works to Prevent Violence Against Women and Girls Global Program supported HTAC to build more rigorous scientific evidence on the efficacy of its programming through an evaluation of a two-year long project implementation in Jawzjan province. This paper presents the endline findings of this evaluation.

## Intervention background

HTAC’s peace education has been developed and implemented in two major phases. The first phase consisted, in 2002, of the development of a six-week school-based program based on an illustrated storybook. This storybook and accompanying resources were developed in English in partnership with McMaster University’s Center for Peace Studies with supervision from an Afghan-Canadian scholar to ensure that the content of the storybook was rooted in Afghan culture, values and experiences. The storybook was then translated into Dari and Pashto and reviewed by other Afghan scholars for accuracy and cultural appropriateness [19].

The second phase of HTAC’s peace education programming began in 2011 and consisted of the development of a more comprehensive peace education curriculum for grades 7 to 12 endorsed by the Afghan Ministry of Education (MoE). The curriculum was developed by a team of international peace education advisors who were responsible for developing curriculum content and learning standards according to Afghan culture and traditions. The peace education curriculum was then translated into Dari and reviewed and approved by the Afghan MoE Curriculum Department [19].

In both phases of HTAC’s peace education program, engaging with teachers, parents and other local community members has been a key strategy for ensuring that peace education principles are supported and reinforced at the school, household and wider community levels [19].

HTAC’s intervention under the What Works to Prevent Violence Against Women and Girls Global Program had four major objectives:

1. Reduce fighting and aggressive behaviour among Afghan boys by encouraging critical reflections and behaviour change that will lead them to reject violence and adopt the principles of peaceful, everyday living;
2. Increase the use of non-violent conflict resolution methods in homes, thereby reducing abuse and threatening behaviour towards women and girls;
3. Teach and motivate male leaders to respect and value women by including them in local councils and supporting their involvement in decision making;
4. Educate women about their rights and protections and empower them to take a more active role in local community affairs.

**Fig 1:**
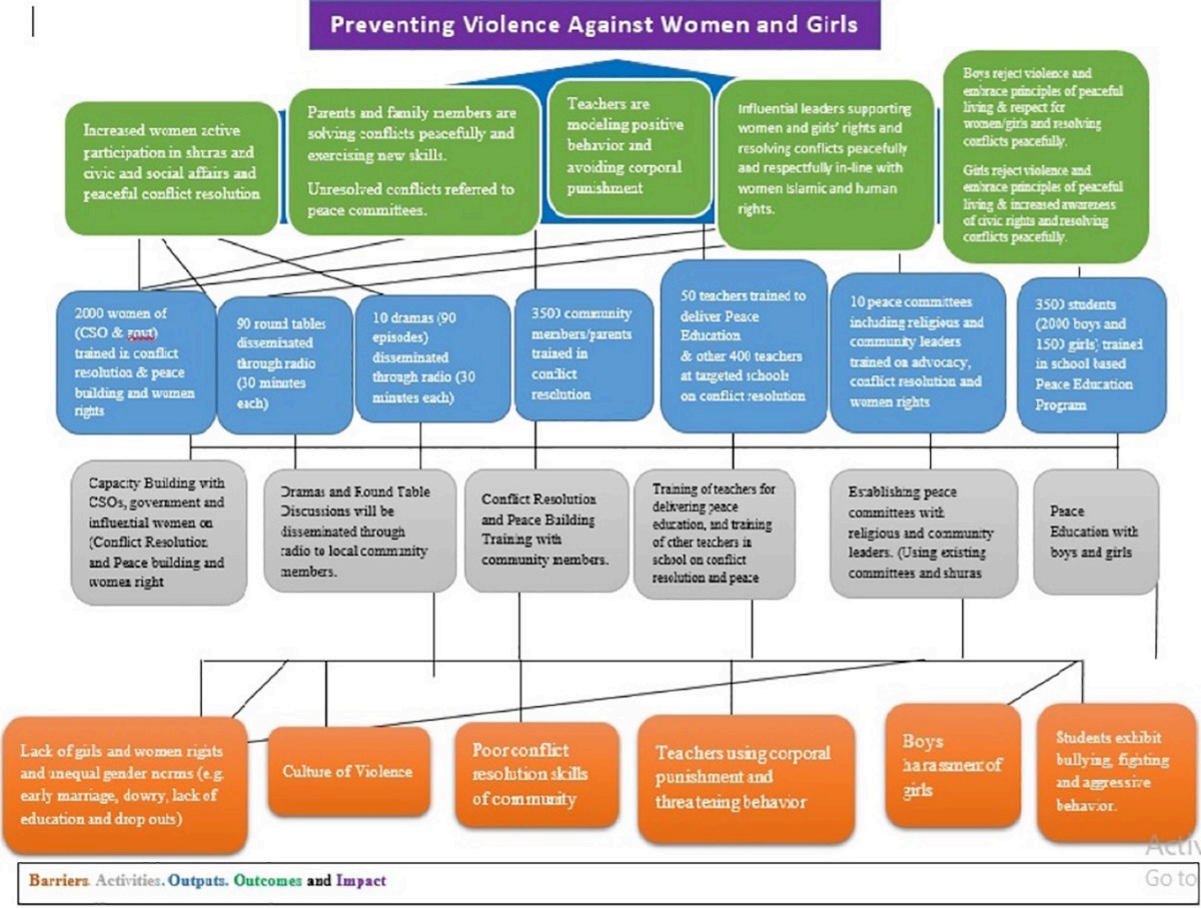
HTAC's theory of change.

In order to achieve these objectives, HTAC’s intervention included a range of activities at the school and community levels and targeted a range of important stakeholders, including children, teachers, parents, community and religious leaders, civil society organisations (CSOs) and influential women (government officials and human rights activists). These activities were implemented in 20 schools (10 boys and 10 girls schools) and their corresponding ten communities in four districts of Jawzjan province. A key focus of the intervention was that activities were implemented intensively in 10 communities among various beneficiaries and stakeholders, comprising a whole community approach. HTAC’s theory of change is depicted in Fig 1.

### Peace education program

HTAC’s Peace Education program ran for two years with the aim of enrolling one cohort of students into the program who would attend peace education classes for the two-year period. HTAC enrolled 2000 boys and 1500 girls (a total of 3500 students) into this cohort. Due to school drop out and migration or displacement linked to conflict or other factors, there was some attrition of students enrolled in peace education classes with 233 dropping out of school after the first year of the peace education program. Consequently, where necessary, HTAC enrolled new students into the second year of peace education classes to maintain an enrolment of 3500 students. Peace education training was delivered either before or after official school hours to students from grades seven and eight (in the first year) and grades eight and nine (in the second year). The peace education curriculum consisted of ninety-nine 35-minute lessons over the two-year period: 33 lessons in the first year (one lesson per week) and 66 lessons in the second year (two lessons per week). HTAC’s peace education curriculum was designed to be delivered over three years with 33 lessons per year; however, since the intervention only had a two-year time span for this particular evaluation, 66 lessons were covered in the second year. The curriculum covered a range of topics, including peaceful, non-violent conflict resolution methods, positive role modeling skills, tolerance for others, respect for women and girls, and rejecting violence and embracing the principles of peaceful everyday living. Learning methods included helping children to understand conflict, deal with grief, loss and anger, and use active communication through mediation, collaborative problem solving and community leadership. Although there was a strong emphasis on boys developing respectful attitudes and behaviours towards women and girls, the peace education curriculum targeted both male and female students in sex-segregated schools.

### Peace education with teachers

Fifty teachers (27 female and 23 male) were trained to deliver the peace education curriculum to students in target schools. Teachers were selected through communication with the Department of Education in Jawzjan province, with key selection criteria being that teachers should have completed Grade 14 (a two year vocational education certificate in teaching) and be teaching social subjects such as Dari, Pashto or History. Teachers participated in a five-day training in how to deliver the peace education curriculum, including a background to peace building and conflict resolution through peace education, the content of the curriculum, how to use the teacher instruction manual and key methodologies for delivering lessons with students. Training methods used with teachers included role play, group work, discussions and practice whereby teachers were asked to deliver lessons to other teachers in the training to ensure there was consistency in the application of teaching methodologies. HTAC also implemented refresher trainings when project monitoring data suggested that teachers required additional support in following the teacher instruction manual or where inconsistent teaching methodologies were observed. There was some attrition of teachers from the program, with new teachers subsequently being trained to facilitate peace education classes. Given that peace education classes were delivered outside of school hours, teachers were provided with monthly stipends to teach the peace education classes in order to provide compensation for their time. In addition, 400 teachers (300 female and 100 male) in intervention schools participated in training on conflict resolution and peace building in order to build their skills in role modeling positive behaviours with students and creating a safe, non-violent classroom environment.

### Conflict resolution, peace building and women’s rights training

HTAC implemented conflict resolution and peace building and advocacy training with a range of different local community actors, including parents and community and religious leaders. A total of 1507 mothers and 1993 fathers (i.e. parents of students participating in peace education classes), and 150 religious and community leaders (30 female and 120 male) participated in the training across the 10 communities. Training sessions covered topics such as peace-building principles and mediation as preferable ways to resolve conflicts and disputes non-violently, how Islam supports the rights and protections of women and girls, and how the involvement of women in community affairs facilitates safer, more secure and prosperous communities.

### Capacity building of women

HTAC sees Civil Society Organisations (CSOs), and particularly women’s rights CSOs, as being key actors in the prevention of violence and support of women’s rights. Consequently, HTAC built the capacity of representatives of women’s CSOs and staff from government departments. A total of 2000 CSO representatives and government officials were trained in 80 training sessions that covered skills in non-violent conflict resolution and mediation, strategies to identify conflict and manage conflicts before they happen, knowledge about women’s constitutional rights and protections, and skills to support their meaningful participation in local civic affairs and community councils.

### Peace committees

HTAC established 10 peace committees across the communities in which the program was implemented. These committees consisted of members from existing community shuras (councils) or community development councils (CDCs). HTAC conducted training with peace committees on conflict resolution and strategies to identify conflicts and prevent them from happening, peace building, advocacy and women’s rights, and skills in mediating and resolving community conflicts related to women and girls (for instance cases involving violence against women). These committees were responsible for responding to community conflicts and disputes, particularly those related to women and children, and resolving them through methods that are in line with HTAC’s peace building and conflict resolution methods and training curriculum.

### Radio messaging

In order also to lengthen the reach of HTAC’s activities into communities in which they were not working directly, HTAC implemented two radio-messaging activities. The first radio messaging activity consisted of 90 weekly radio round table discussions that typically involved between three and five participants (e.g. religious leaders, influential women, women’s CSO members and other civil society activists, and government officials). These participants discussed set topics related to the rights of women and girls (e.g. early and forced marriage, violence against women and girls, lack of girls’ access to education, child protection, women’s empowerment and the role of women in development and economic activities). Round table discussions were pre-recorded before being aired on the radio in order to ensure that discussions did not reproduce harmful messages that could work against the aims of the program. In order to encourage community members to access the radio round table discussions, HTAC assembled and helped to facilitate community round table listener groups, comprising parents and other community members who would congregate in an available space to listen to the radio and discuss the content.

The second radio messaging activity consisted of airing 90 episodes of scripted radio dramas. Each episode dealt with a different issue related to conflict and violence against women; for instance, not allowing girls to go to school or favouring the education of boys, denying women’s rights to property and inheritance, and the prevalence of intimate partner violence against women in Afghan families.

## Methods

### Setting

This evaluation was conducted in three districts of Jawzjan province (Sheberghan, Aqcha and Faizabad), in 11 secondary schools receiving HTAC’s intervention. Six of these schools, all in Sheberghan district, were located in urban settings, with the remaining five schools (in Aqcha and Faizabad) located in rural settings. The average class size in target schools is 30 students, and the proportion of female teachers is higher than for male teachers in both boys and girls schools.

Jawzjan province is located in the north of Afghanistan, along the border with Turkmenistan; however, all three districts are located in the south of the province, with borders shared with a number of other provinces. Jawzjan province is ethnically and linguistically diverse, with Dari commonly spoken but with large populations of native Turkmen and Uzbek speakers. Security was highly unstable in Jawzjan during the time that HTAC’s intervention and this evaluation were conducted, with anti-government groups particularly active in Aqcha district.

### Participants

Research was conducted with students (boys and girls) in 11 secondary schools receiving HTAC’s intervention (seven girls’ and four boys’ schools). The target sample was 720 students (360 boys and 360 girls). There was no pre-baseline pilot to establish peer violence prevalence, nor any other available peer violence prevalence data at the time of research design, which precluded the development of a sample size required to measure a minimum detectable effect. Consequently, a sample size of 720 students was selected as an estimate to assess reduction in peer violence among children at each evaluation point. It was anticipated that a sample size of 720 children would be adequate for this. There were some variations in the student samples at each evaluation point: 350 boys and 420 girls at baseline, 356 boys and 364 girls at midline, and 361 boys and 373 girls at endline.

### Research design

The data were derived from an interrupted time series study with three data collection points. Baseline data collection was conducted in October and November 2016, midline data collection was conducted six months post-baseline (May 2017) and endline data collection was conducted 12 months post-baseline (November 2017). An independent research organisation was contracted to conduct the data collection. The original research design involved data collection in all 20 schools in which HTAC implemented its peace education program. However, monitoring and data quality control processes implemented in the first fielding of baseline data collection identified a number of challenges, including inadequate enumerator training, poor enumerator fieldwork practice and coordination with schools, and poor data quality. Consequently, a new research organisation was contracted and the baseline was conducted a second time with additional measures implemented to ensure data quality, including a senior researcher overseeing and leading enumerator training, closer coordination with schools, the development of more structured fieldwork protocols and intensive data collection monitoring in the field. The second baseline was implemented approximately seven months after HTAC began its programming, in March 2016. During the first baseline, nine schools had been sampled before decisions were made to terminate the data collection and the remaining 11 schools were later sampled in the second baseline.

The research design was cross-sectional, with a sample of students surveyed at each evaluation point. At baseline, students were in grades 7 and 8, and the same grade cohorts were sampled at midline and endline in grades 8 and 9. Children participating in HTAC’s peace education classes and their parents had already provided consent for children to participate in the peace education intervention prior to baseline data collection. Parents and children were then requested to provide informed consent to participate in the research, and samples of students at each evaluation point were obtained from a list of children with confirmed personal and parental consent to participate in the research.

According to the research design, students would not be tracked and resampled for data collection but, rather, a new random sample of students would be taken at each evaluation point such that each sample would comprise different students. This was not possible to implement for a number of reasons. The requirement to redo the baseline data collection in 11 rather than the full 20 schools meant that larger samples of students had to be obtained in each school, limiting the ability to sample three new cross sections of students at each evaluation point given the fixed number of peace education enrolments. This was further complicated by some attrition of students from the peace education program in the second year of the intervention, further reducing the possible pool of students from whom to sample for the evaluation. Consequently, random samples of students were obtained at baseline and midline from the same list of possible students (i.e. those with parental and individual informed consent), with post-field matching identifying that almost 50% of baseline students were also sampled at midline, and the remaining 50% at midline comprising new students. The same students sampled at midline were also sampled at endline, with 100% retention between the midline and endline samples. Despite approximately 50% of the sample being consistent across time, a longitudinal analysis was not possible given that the cross-sectional research design precluded tracking students and, thus, student ID numbers were not linked to corresponding survey data.

Students were interviewed with a questionnaire designed for the study, translated from English to Dari, and administered by trained enumerators. Female enumerators interviewed girls and male enumerators interviewed boys. The student questionnaire was based in part on a questionnaire developed in English, piloted in Pakistan for research conducted with children of a similar age [21] and then piloted in Afghanistan, with subsequent minor modifications made to ensure the tool was age and culturally appropriate.

### Measures

Students were asked about their social and demographic details, including age, school grade, total household members and number of siblings (brothers and sisters). Socio-economic status was assessed through two food insecurity questions measuring the frequency of going to school without breakfast and to bed without dinner. Question values ranged between zero and three (0 = never, 1 = once, 2 = two or three times, and 3 = four or more times) and values were summed to give a food insecurity/hunger score (higher values indicate more food insecurity). Students’ disability was measured through five questions derived from the Washington Group on Disability Scale, including difficulty: seeing, hearing, walking or climbing, remembering/concentrating, or speaking. Students are coded as having a disability if they responded ‘a lot of difficulty’ or ‘cannot do at all’ to any of the five questions, in line with Washington Group analytic guidelines [22].

School performance was measured with two key indicators: (1) number of days absent from school in the past month, and (2) learning achievements. The second indicator was measured through simple literacy and numeracy tests administered during the survey, including reading one line of the questionnaire, and completing three mathematics equations (16+4, 7+3, 25/5). For literacy, learners were coded on a scale of one to four, as not able to read at all, reading with difficulty, reading with little difficulty, or reading fluently. For numeracy, learners were coded on a scale of one to four, as not numerate at all, adding with difficulty, adding with ease or dividing with ease. Literacy and numeracy items were summed to give an overall learning achievement score.

There were a number of key outcomes linked to the peace education component of the intervention. There were three key outcome variables related to children’s experience of violence: peer violence victimisation, peer violence perpetration, and corporal punishment by teachers at school. Peer violence victimisation was measured through The Peer Victimization Scale [23], consisting of 16-items (Table 1) and designed for young people aged 11 to 17. Students were asked over the past month how often (i.e., never, once, a few times (2–3) or many times (4 or more)) an event happened to them. Peer victimisation was recorded if there was more than one episode or type of violence. A Peer Perpetration Scale was developed based on the Peer Victimization Scale by using the same scale items but with the wording adjusted to measure perpetration and the same approach to recording occurrence of peer perpetration. Students’ experience of corporal punishment by teachers at school was measured through six questions (Table 1) and a variable was derived as a measure of any experience of corporal punishment at school in the past month.

**Table 1:** Student questionnaire items used to construct outcomes.

	Indicator	Respondents	Items in composite indices	Cronbach’s alpha	Expected direction of change
Peer violence	% of students who are victimized by peers	Boys, girls	Peer Victimisation Scale. 16 items (four subscales, each with 4 questions) assessing peer victimisation: (1) Physical victimization (tripped me to make me fall, pushed me to hurt me, hurt me physically, beat me so badly that I was injured) (2) Verbal victimization (called me bad names, made fun of me because of my appearance, made fun of me for some reason apart from my appearance, swore at me) (3) Social manipulation (tried to get me in trouble with my friends, refused to talk to me, made other people not talk to me, tried to make other children turn against me) (4) Property attacks in the previous 4 weeks (deliberately broke something that belongs to me, stole something from me, tried to take something of mine without permission or deliberately damaged something of mine).	N/A	Decrease
	% of students who perpetrate violence against peers	Boys, girls	Peer Perpetration Scale. Modified from the Peer Victimisation Scale, with the same 16 peer victimization items and subscales with wording adjusted to measure perpetration.	N/A	Decrease
School-based corporal punishment	% of students who experienced corporal punishment at school in the past month	Boys, girls	Any instance of corporal punishment by teachers at school in past month, including: being slapped, hit, beaten or physically punished; having ears twisted; made to stand on a bench; made to run around as punishment; made to kneel down; or hit with a stick, whip or other object.	N/A	Decrease
Gender attitudes	Mean gender equitable attitudes score	Boys, girls	Gender attitudes scale with 13 items. *Four statements* related to women’s participation in everyday activities: weddings, neighborhood events, skills training and income generating activities. *Nine statements* related to women’s and girls participation and decision making: I think girls in our family should go to school; I think the husbands in my family should give permission to their wives to go to the clinic; I think the husbands in the family should listen to their wives’ opinion on schooling; I think the wives in my family should have a say in how money in their family is spent; I think the wives in my family should be able to ask a religious scholar about issues; I think the husbands in my family should respect the opinion of their wives on matters related to income generating work; I think a husband in my family should be kind and caring toward the women in his family; I think that the wives in my family should always obey their husbands; and I think that if a wife in my family does something wrong her husband has the right to punish her.	Baseline: 0.84 Midline: 0.84 Endline: 0.87	Increase
Attitudes to child punishment	Mean child punishment scores	Boys, girls	Attitudes towards child punishment and fighting scale with five items: I think that if a child disobeys their parents they should be beaten; I think that if a child gets into fights their parents should beat them; I think that if a child talks back to their parents they should be punished by being beaten; I think a child who misbehaves at school should be beaten; and I think that if a child hurts me I should hurt them back.	Baseline: 0.91 Midline: 0.88 Endline: 0.92	Decrease
Depression	Mean CDI-II depression scores	Boys, girls	28 items related to children’s self-rated feelings of sadness, self-hate, negative self-image, suicidal ideation, loneliness, fatigue etc. in the last two weeks.	Baseline: 0.81 Midline: 0.79 Endline: 0.82	Decrease
Hope	Mean hope scores	Boys, girls	Six items: I can think of many ways to get out of a difficult situation; I put lots of energy into pursuing my goals; there are lots of ways around any problem; I can think of many ways to get the things in life that are important to me; even when others get discouraged, I know I can find a way to solve the problem; and I meet the goals that I set for myself.	Baseline: 0.74 Midline: 0.84 Endline: 0.86	Increase

The study included two scales related to attitudes and perceptions: a gender equitable attitudes scale, and an attitudes to child punishment scale. The gender attitudes scale, consisting of 13 items (Table 1) measured on a 4-point Likert response scale (strongly disagree to strongly agree), has response values ranging from zero to three. Items were summed to obtain a gender (equitable) attitudes score with a possible range of between zero and 39. The attitudes towards child punishment scale contains five items measured on a 4-point Likert response scale (strongly disagree to strongly agree), with values from zero to three. All items were summed to obtain a child punishment attitudes score with a possible range of between zero and 18, with higher scores indicating more support for violent practices.

Outcomes related to psychosocial wellbeing include a depression scale and a hope scale. Students’ experience of depression was measured through a modified Children’s Depression Inventory (CDI—2) [24], which includes 28 items (Table 1). Item values range from zero to two with higher values indicating more severity of depressive symptoms. Values for each item are summed to obtain a depression score, which is then converted to a sex-specific and age-adjusted Total score (T-score) with a possible range of 40 to 90. Higher scores indicate more depressive symptoms with 65 as the cut off for depression. Students’ sense of hope was measured through a six-item scale modified from Snyder et al.’s [25] Hope Scale (Table 1). Items are measured on a 4-point Likert response scale (strongly disagree to strongly agree), with values from zero to three summed to obtain a hope score with a possible range of between zero and 18, with higher scores indicating more hope.

Cronbach’s alpha for gender attitudes scores, attitudes to child punishment scores, depression scores and hope scores are included in Table 1 for baseline, midline and endline evaluation points. For all scales at all evaluation points, alpha levels were above 0.70 and thus scales were deemed to have good internal consistency.

In addition to the key outcomes outlined above, students were asked a series of questions about their observation and experience of violence at home in order to gather information that may shed light on family and community-level change due to the intervention. Students were asked two questions about having observed abuse of their mother in the previous month, one related to physical violence by students’ fathers, and the other about physical violence from any family member. A variable was then derived as a measure of any abuse of students’ mothers in the past month. Students were also asked if they had seen or heard their father fighting physically with another man in the last month. Students’ experience of corporal punishment at home was measured through two items: how often in the past month they had been slapped, hit, beaten or physically punished by a parent; and whether they had been beaten so hard at home in the past month that they were injured. A variable was derived from these two questions as a measure of any experience of physical violence at home in the past month.

At midline, due to some delays in the fieldwork, the data collection crossed into Ramadan by two days, and 28 students (from one boys’ school in particular) were sampled during these two days. This led to some data variations for a few variables, including disability; however, there was no impact on any of the primary violence outcomes of this study.

### Ethics

This study was approved by the Institutional Review Board (IRB) of the Ministry of Public Health, Afghanistan, and the Ethics Committee of the South African Medical Research Council. HTAC obtained all necessary permissions from the Provincial Education Directorate in Jawzjan province and schools participating in the study. HTAC provided a verbal description of the study to school principals, teachers and students, and consent forms and information sheets were translated into Dari and given to children to take home and give to their parents. Only children with signed consent forms from their parents and who gave verbal consent themselves were recruited into the study.

### Data analysis

Data collected at the three evaluation points were merged into a long format, identified by data collection wave. All analysis took into account the study design by adjusting for clustering of participants at school level. The analysis was done on Intention to Treat (ITT) basis—we included all participants sampled irrespective of their exposure to the intervention. We compared demographic characteristics of participants sampled at each data collection round.

With very minimal or no missing data in the outcome, no imputations were performed on any of the outcomes. Descriptive statistics were used to describe the proportion of participants who reported each characteristic or behaviour at each evaluation point. Additive scores were derived from scale items for scales such as gender attitudes and attitudes associated with child punishment. We used histogram and normality tests to check the distributions of all outcomes at each data collection point.

Continuous outcomes were summarized using means and the 95% upper confidence limit (UCL) and lower confidence limit (LCL) of the mean for each data collection round. Dichotomous outcomes were summarized using proportions and their respective UCLs and LCLs. For both continuous and dichotomous outcomes, we used Taylor linearization to calculate standard errors for the estimates. Temporal trend tests were performed on all the outcomes to assess if there was any monotonic change in the outcomes over time. All tests were done at 5% significance level and all analyses were done using STATA version 14.

## Results

Students’ social and demographic characteristics vary only slightly across evaluation points. There are some larger variations in students’ age categories, with the sample of both boys and girls getting older across evaluation points, which is expected given that students have aged one year between baseline and endline (Table 2). For both boys and girls, the proportion of children who are food insecure is much smaller at endline than at baseline; however, at all evaluation points, girls are more food insecure than boys. The proportion of boys who are categorized as having a disability increases substantially at midline and then drops to half the baseline proportion at endline. The large increase in boys’ disability at midline can be largely accounted for by difficulties with memory and concentration. Given that food insecurity drops for boys across evaluation points, it is unlikely that memory and concentration difficulties are due to hunger. It is much more likely that boys’ memory and concentration difficulties at midline is due to hunger as a result of fasting for Ramadan rather than food insecurity, and this is most visible in the one boys’ school in particular that was sampled at the end of the data collection period during Ramadan. Girls’ disability status also changes over time, although we see a reduction in reported difficulties across all three evaluation points.

**Table 2:** Socio-demographic characteristics of the learners.

	Baseline (boys: n = 350, girls: n = 420)			Midline (boys: n = 356, girls: n = 364)			Endline (boys: n = 361, girls: n = 373)		
**BOYS**	%/ mean	LCL	UCL	%/ mean	LCL	UCL	%/ mean	LCL	UCL
Age group									
< = 13yrs	21.4	9.5	41.4	7.0	1.2	31.9	4.4	0.8	20.3
14yrs	32.6	23.2	43.5	21.1	11.6	35.2	18.3	13.1	24.9
15yrs	26.3	11.1	50.5	30.3	24.7	36.6	31.0	22.6	41.0
> = 16yrs	19.7	16.0	24.1	41.6	24.2	61.4	46.3	35.7	57.1
Food insecure (%)	16	3.6	49.4	4.2	0.5	27.2	6.9	0.4	56.8
Disability (%)	12.9	4	34.3	22.8	2.2	79.8	6.6	1.5	24.4
**GIRLS**									
Age group									
< = 13yrs	28.8	22.4	36.1	12.6	6.7	22.5	9.7	5.3	17
14yrs	33.8	26.9	41.5	25.3	16.2	37.2	27.1	16.3	41.4
15yrs	20.7	14.8	28.1	34.6	27.4	42.6	26.3	19	35.1
> = 16yrs	16.7	8.8	29.2	27.5	14.8	45.3	37	21.8	55.3
Food insecure (%)	17.6	9.4	30.7	7.4	4.6	11.7	8.6	5.9	12.3
Disability (%)	22.1	10.7	40.4	15.1	5.6	34.9	11.0	3.8	27.9

LCL: 95% lower confidence limit for mean/percentage at each data collection point. UCL: 95% upper confidence limit for mean/percentage at each data collection point.

Children’s school performance, measured through basic literacy and numeracy tests, indicate no difference between baseline and endline, for either boys or girls (Table 3). School attendance for boys is slightly poorer at endline than at baseline, although this reduction in attendance is not significant. In contrast, girls’ attendance has improved significantly between baseline and endline. The gender differences in school attendance are also visible in students’ reports of attendance in peace education classes, whereby a larger proportion of girls reported attending peace education classes every week in the last year at both midline (83.2%) and endline (81%) than boys (63.5% at midline and 71.8% at endline). Although at baseline illness was a common reason for missing school, significantly fewer boys and girls are missing school due to illness at endline. At endline, fewer girls are missing school due to working at home and more boys are missing school due to working at home or working for money. The findings change direction at midline, where we see fewer boys working at home and more girls working at home. This may be partly due to a small portion of the midline data collection happening during Ramadan, where girls are typically required to assist their female family members more with domestic chores and cooking for Iftar (the breaking of the fast). At midline, there was an increase in boys and girls stating that they missed school for other reasons. Although the survey did not clarify which other reasons were relevant, it may due to Ramadan, where children of lower-secondary age are typically fasting, which may impact negatively on attendance. For both boys and girls, at endline there is a highly significant reduction in children’s reports of corporal punishment by teachers in the month prior to data collection, and prevalence of corporal punishment has more than halved between baseline and endline for girls.

**Table 3:** School environment outcomes for learners.

	Baseline (boys: n = 350, girls: n = 420)			Midline (boys: n = 356, girls: n = 364)			Endline (boys: n = 361, girls: n = 373)			
**Boys**	%/ mean	LCL	UCL	%/ mean	LCL	UCL	%/ mean	LCL	UCL	sig
Average number of school days missed	2.4	2.0	2.9	2.6	1.5	3.7	3.0	1.5	4.5	0.353
School performance (mean)	6.2	5.1	7.4	5.9	4.1	7.7	6.2	5.0	7.4	0.209
Reasons for Missing school										
Illness	22.0	5.6	57.3	19.1	7.0	42.6	14.7	13.0	16.5	0.012
Helping at home	29.7	19.0	43.2	21.6	6.8	51.0	33.2	17.5	53.9	0.258
Working to earn money	9.1	2.0	33.0	11.5	3.5	32.1	14.4	3.8	42.0	0.029
Other reasons	27.7	16.0	43.5	36.5	13.1	68.7	12.7	4.5	31.3	<0.001
Corporal punishment experience	43.7	9.7	84.9	33.4	17.3	62.9	27.2	10.4	54.5	<0.001
**Girls**										
Average number of school days missed	2.2	1.9	2.5	1.7	1.4	2.0	1.4	0.9	1.9	<0.001
School performance (mean)	6.5	5.9	7.1	6.6	5.9	7.3	6.5	5.8	7.2	0.969
Reasons for Missing school										
Illness	31.7	23.3	41.4	22.0	17.4	27.4	20.1	14.8	26.8	<0.001
Helping at home	37.4	23.0	54.4	39.8	28.6	52.3	28.7	13.4	51.2	0.013
Working to earn money	1.0	0.3	3.4	0.3	0	3.1	0.3	0	3.1	0.173
Other reasons	11.9	4.8	26.6	15.9	6.7	33.2	8.0	3.5	17.3	0.114
Corporal punishment experience	35.0	23.6	48.5	20.3	13.2	30.1	14.2	18.6	29.7	<0.001

LCL: 95% lower confidence limit for mean/percentage at each data collection point. UCL: 95% upper confidence limit for mean/percentage at each data collection point. sig: p-value for the temporal trend test on monotonic change.

There are no significant differences between baseline and endline in relation to boys’ observation of their fathers fighting or hitting boys’ mothers (Table 4). However, there is a significant reduction at endline in the proportion of boys who witnessed their mothers being beaten by other family members, although the proportion is low at all evaluation points. Significantly fewer girls at endline observed their fathers fighting with another man, or observed their mothers being physically beaten by any family member. There was a large and significant reduction in boys’ and girls’ reports of experiencing physical punishment at home in the past month. Furthermore, both boys and girls have significantly more equitable gender attitudes and significantly less violence-supportive attitudes at endline than at baseline.

**Table 4:** Violence at home and gender attitudes.

	Baseline (boys: n = 350, girls: n = 420)			Midline (boys: n = 356, girls: n = 364)			Endline (boys: n = 361, girls: n = 373)			
**Boys**	%/ mean	LCL	UCL	%/ mean	LCL	UCL	%/ mean	LCL	UCL	sig
Seen or heard father fight with another man in last 4 weeks	5.1	1	23.3	2.5	0.8	8	3.6	0.4	27.4	0.285
Seen or heard father hit mother in last 4 weeks	1.4	0.2	8.7	3.4	0.2	39.4	3.3	0.5	19.5	0.122
Seen or heard mother beaten by other relatives in last 4 weeks	2.3	0.2	18.4	0.8	0.3	2.4	0.3	0	6.3	0.011
Any abuse of mother in last 4 weeks	3.4	0.5	19.3	3.9	0.4	30.1	3.3	0.5	19.5	0.937
Experienced physical punishment at home in last 4 weeks	16.6	5	42.7	14.9	4.1	41.7	4.7	1.3	15.3	<0.001
Gender equitable attitudes mean score (higher = more equitable)	29.3	25.2	33.3	32.4	27.1	37.8	32.1	28.2	36.0	<0.001
Attitudes to corporal punishment mean score (higher = less support for CP)	13.9	9.7	18.0	15.7	10.8	20.5	16.9	13.5	20.3	<0.001
**Girls**										
Seen or heard father fight with another man in last 4 weeks	14.5	4.7	36.8	6	3.6	10.1	3.8	1.7	8	<0.001
Seen or heard father hit mother in last 4 weeks	5.2	2.3	11.4	1.4	0.3	5.7	2.4	1.2	4.8	0.018
Seen or heard mother beaten by other relatives in last 4 weeks	2.1	1.1	4.2	2.2	1.1	4.5	0.8	0.3	2.5	0.157
Any abuse of mother in last 4 weeks	6.7	3.4	12.7	2.7	1.3	5.7	2.7	1.3	5.6	0.004
Experienced physical punishment at home in last 4 weeks	20.0	7.0	45.4	8.5	4.7	15.1	2.7	1.6	4.4	<0.001
Gender equitable attitudes mean score (higher = more equitable)	30.8	28.0	33.7	29.5	28.3	30.6	34.3	30.7	38.0	<0.001
Attitudes to corporal punishment mean score (higher = less support for CP)	17.1	15.2	19.0	16.7	15.5	18.0	18.1	16.5	19.7	<0.001

LCL: 95% lower confidence limit for mean/percentage at each data collection point. UCL: 95% upper confidence limit for mean/percentage at each data collection point. sig: p-value for the temporal trend test on monotonic change.

There is a significant decrease between baseline and endline in students’ experiences of peer violence perpetration and victimisation (Table 5). The proportions of girls and boys who experienced peer violence victimisation or who perpetrated violence against their peers in the past month have dropped by approximately a half. The reduction in peer violence between baseline and endline is particularly large among non-perpetrating boys and girls who reported victimisation. Boys and girls also have significantly lower depression scores at endline. Further, the proportion of students classified as depressed according to a CDI T-Score cut off of 65 reduced by almost a half (from 27.8% at baseline to 15.1% at endline), with the reduction proportionately higher for girls (from 16.2% at baseline to 5.7% at endline) than boys (from 41.7% at baseline to 24.9% at endline). Girls have higher hope scores although we don’t see the same for boys, whose hope scores have increased only slightly.

**Table 5:** Peer violence perpetration and/or victimization, depression and hope.

	Baseline (boys: n = 350, girls: n = 420)			Midline (boys: n = 356, girls: n = 364)			Endline (boys: n = 361, girls: n = 373)			
**Boys**	%/ mean	LCL	UCL	%/ mean	LCL	UCL	%/ mean	LCL	UCL	sig
Any peer violence perpetration or victimization in last 4 weeks	57.7	19.4	88.6	43.5	10.9	83	29.1	7.5	67.5	<0.001
Any peer violence perpetration in last 4 weeks (with or without victimisation)	31.7	11.9	61.4	29.5	3.6	82.4	13.6	2.6	48.5	<0.001
Any peer violence victimization in last 4 weeks (with or without perpetration)	49.7	15.2	84.5	35.4	12.9	66.9	25.2	5.6	65.6	<0.001
Among non-perpetrators, any peer violence victimization in last 4 weeks	42.9	11.1	81.9	34.3	4.2	86.3	16.1	2.6	58.1	<0.001
Depression score (mean)	63.7	47.8	79.6	57.8	50.3	65.2	57.7	47.3	68	<0.001
Hope score (mean)	14.2	11.8	16.7	15.3	11.5	19.0	15.0	13.8	16.2	
**Girls**										
Any peer violence perpetration or victimization in last 4 weeks	46.9	29	65.7	41.5	22.4	63.5	23.6	11.9	41.3	<0.001
Any peer violence perpetration in last 4 weeks (with or without victimisation)	17.6	9.8	29.7	13.2	7.3	22.6	7.2	3.1	16.2	<0.001
Any peer violence victimization in last 4 weeks (with or without perpetration)	43.3	25.5	63.1	38.7	19.7	62	21.7	10.7	39	<0.001
Among non-perpetrators, any peer violence victimization in last 4 weeks	24.9	12.1	44.5	18.4	8.5	35.3	8.7	3.3	20.9	<0.001
Depression score (mean)	57.7	54.4	61.1	55.6	50.8	60.4	52.1	48.8	55.5	<0.001
Hope score (mean)	13.5	12.4	14.6	13.7	13.2	14.2	16.0	14.6	17.4	

LCL: 95% lower confidence limit for mean/percentage at each data collection point. UCL: 95% upper confidence limit for mean/percentage at each data collection point. sig: p-value for the temporal trend test on monotonic change.

## Discussion

HTAC’s intervention primarily aims to reduce peer violence and corporal punishment against children at the school level, and reduce violence against women and girls at the broader community level. The evaluation of HTAC’s intervention suggests that it has been very successful in achieving these aims. Although high levels of peer violence victimisation and perpetration were observed at baseline, particularly for boys, there has been more than a 50% reduction in both peer violence victimisation and perpetration by the endline evaluation point, and this is true for both boys and girls. These findings suggest that peace education can be an effective approach for reducing interpersonal violence and promoting peaceful and respectful conflict resolution among school-aged children. The results of the evaluation also show that students’ reports of corporal punishment by teachers at school have reduced significantly. For girls, experience of corporal punishment has reduced by more than 50% and for boys it has reduced by more than a third.

Although school-based interventions are appropriate for reducing children’s peer violence and experience of corporal punishment at school, they are not necessarily effective in reducing violence against women and children at the household level. Indeed, the baseline evaluation findings suggested that there was a strong association between children’s experience of physical violence or witnessing physical violence at home, and their perpetration of violence against peers [24]. Consequently, HTAC implements a number of activities at the community level to raise awareness of women’s and girls’ rights and the prevention of violence against women, including: peaceful conflict resolution training with community members, and community training and radio messaging on women’s and girls’ rights and violence prevention. The evaluation suggests that these activities have been successful in reducing violence at the household level. Both boys and girls reported experiencing significantly less physical punishment at home, and observing less family violence at home. Significantly fewer girls observed their fathers fighting at endline, and significantly fewer girls and boys observed their mother being beaten at home. It is possible that reported behaviour change at the household level was partly due to children sharing their learning about peaceful conflict resolution with family members and thus directly influencing their behaviour. However, given that parents and other community members participated in specific and targeted activities associated with violence prevention (i.e. women’s rights training and listening to radio round tables and radio dramas), it seems more likely that these activities would be associated with behaviour change within the family. However, as noted further below, this is difficult to corroborate given that the pathway to parental behaviour change cannot be confirmed from this study due to data not being collected from parents or other community members.

The intervention had a positive impact on children’s gender equitable attitudes and violence-supportive attitudes. At baseline, large proportions of children agreed with statements related to women’s and girls’ rights to social and economic participation and decision-making, but many children also agreed with a statement related to men’s punishment of their wives (I think that if a wife in my family does something wrong her husband has the right to punish her). At endline, children’s (both boys’ and girls’) gender equitable attitudes improved significantly, and it was evident that the improvement has happened in particular for responses to the statement presented above, with 47.6% of students agreeing with the statement at baseline and less than half this proportion of students (22.1%) agreeing at endline. This suggests that, in particular, children’s attitudes towards violence against women improved. The evaluation shows that children’s attitudes towards the physical punishment of children had also improved, with violence-supportive attitudes reducing among both boys and girls by the end of the intervention. The findings indicate that children were less supportive of violence against children at endline on a number of key measures, including violence perpetrated by parents against children, by teachers against students, and by children against other children.

Students’ psychosocial wellbeing improved over the course of the intervention. Girls were more hopeful at endline than at baseline, although the same was not found for boys. Both boys and girls at baseline had mean depression scores below the cut-off of 65, although the mean score for boys (63.5) was close to the cut off. Both boys’ and girls’ depression scores were significantly lower at endline and a smaller proportion of students at endline were classified as depressed, with a 65% reduction in the proportion of girls identified as depressed and a 40% reduction for boys. The endline findings for depression may be related to the reduction in peer violence experienced by students, particularly given that the baseline results indicated a significant relationship between peer violence (both victimisation and perpetration) and depressive symptoms among girls [26].

Students’ school performance, measured through basic literacy and numeracy tests, showed no change in the one year between baseline and endline, suggesting that children were struggling with learning achievements. This is consistent with existing evidence in Afghanistan that suggests that by grade 6 few children are able to perform adequately in grade-level literacy and numeracy tasks [27]. The evaluation did find, however, that girls’ school attendance had improved at endline. Given the baseline relationship observed between girls’ peer violence victimisation and poorer attendance [26], it is likely that a reduction in peer violence at endline has enabled this improvement in girls’ attendance.

There are some irregular patterns in the data, including students’ increased self-reported disabilities and a large decrease in school attendance between baseline and midline, with the findings moving in the expected directions between midline and endline. The increase in disability is mainly in the area of concentration and memory. Given that children’s food insecurity decreases significantly across evaluation time points, it is unlikely that difficulties with memory and concentration are a result of hunger from food insecurity. When examining in which locations the increase in disability occurred, it is evident that it is concentrated in the boys school that was partially sampled in the first week of Ramadan. It is likely that memory and concentration difficulties are in fact a result of hunger, albeit not from food insecurity, because students were fasting. Although the decrease in attendance between baseline and midline is not concentrated in any particular schools, it does appear that attendance has dropped prior to the onset of Ramadan, potentially due to children’s requirements to assist households to prepare for Ramadan.

There are a number of strengths and limitations of the research. Although there was no control group to test a counterfactual, the study employed an interrupted time series design with three data collection points, which is an appropriate design for an intervention that has not been previously evaluated. This design allowed us to analyse trends across time points, showing consistency of impact. Another strength of the design is that data collection was fielded in a large number of schools across three different districts, allowing the study to capture children’s experiences from ethnically and linguistically diverse parts of the province where HTAC implemented its intervention.

A key limitation of the research is that the researchers were unable to measure attendance at the intervention and so we have no way of assessing fidelity and dosage. We were also unable to tease out which components may have been more important for impact. We hypothesize that the school-based component primarily impacted the children, and the community-intervention the adults, but we did not test this hypothesis. It is possible that the impact on both groups was due to the two components being delivered together in this complex multi-component intervention. A further limitation is that although the finding that children observed significantly less family violence at home (e.g. physical abuse of their mothers) is an important sign of change, we did not interview parents or other household members. Consequently, it is not possible to triangulate these results, in particular, through women’s reports of less domestic violence and so the pathway to parental behaviour change reported by children is not clear and deserves additional attention in future research.

Another limitation of the study is that follow up between baseline and endline time points was only 12 months. This was due to challenges in the baseline data collection and the requirement to field baseline data collection seven months after the start of the intervention. Consequently, it is possible that even more significant change might have been observed if the baseline was completed prior to or at the beginning of the intervention period. Nevertheless, that significant changes were observed in a 12-month period lends some support to the strength of the intervention. A further limitation in the study was the timing of the midline data collection, which, as outlined above, spilled over for two days into the beginning of Ramadan. Data collection during Ramadan can be challenging for a number of reasons, including students having lower school attendance during the Ramadan month, and this may partly explain some of the irregular data found at midline, including for disability and attendance. However, the data collection in Ramadan at midline did not significantly impact on key violence outcomes, including students’ experiences of peer violence and corporal punishment, or observations of family violence.

To our knowledge this is the first time that a peace education program has been evaluated in Afghanistan, with or without the community-based intervention to change harmful social norms and practices related to gender and the use of violence in conflict resolution. The majority of peace education evaluations in other countries have measured the efficacy of conflict resolution curricula to promote safe school environments; however, few peace education programs have additional components that target both children and adults at the school and community levels [15, 16]. This evaluation suggests that conducting peace education with children in schools, alongside components for adults in the community complementing this education with broader messaging about women’s and children’s rights and the prevention of violence, can lead to a reduction in various forms of violence, including children’s peer violence, corporal punishment of children both at school and at home, and in reports of children witnessing domestic violence against women at the household level. This is important as a potential contribution to the broad social goals of building peace communities in conflict ridden Afghanistan, as well the immediate impact on children and women. Further the reduction of violence among children has the potential to reap benefits into the future for reducing violence in their later marital relationships.

## Supporting information

S1 File This file contains the student questionnaire in English. (DOCX)
