# Supplementary material for: What works to prevent violence against children in Afghanistan? Findings of an interrupted time series evaluation of a school-based peace education and community social norms change intervention in Afghanistan
Source: PLoS One. 2019 Aug 6;14(8):e0220614. doi: 10.1371/journal.pone.0220614 (PMC6684089; doi:10.1371/journal.pone.0220614)
Supplement: S1 File — (DOCX) [file pone.0220614.s001.docx]

**STUDENT QUESTIONNAIRE**

**Study ID Number ……………………………**

**Name of school………………………………**

**School ID Number ……………………………**

**Interview round (circle): 2016 2017 2018**

**Date __________________________________**

| **ABOUT YOU** | | | |
| --- | --- | --- | --- |
| 1 | How old are you? | | -----------------------age in years |
| 2 | Boy or a girl?  (Enumerator to complete) | | Boy……………………………….1  Girl ………………………………2 |
| 3 | Your Grade in School ___________________________________ | | |
| 4 | How many people live in your home? | | ____________(number) |
| 5 | How many brothers do you have? | | ____________(number) |
| 6 | How many sisters do you have? | | ____________(number) |
| **SCHOOL** | | | |
| 7 | How many days of school did you miss in the last 4 weeks? Number __________________ | | |
| 8 | The last time you missed a day from school, what was the main reason? | a You were ill …………….….. Yes = 1 No=2  b No money for transport… Yes = 1 No=2  c Helping at home ……….….. Yes = 1 No=2  d Working to earn money…… Yes = 1 No=2  e Afraid to go to school due to violence Yes = 1 No=2  f Did not want to go to school for another reason  Yes = 1 No=2 | |
| 9 | How well can student read?  Tested by interviewer asking child to read line of questionnaire | Cannot read at all ………………………………………..1  Can read with difficulty…………………………………..2  Reads with little difficulty………………………………3  Reads fluently…………………………………………….4 | |
| 10 | How good is student at maths?  Tested by interviewer:  16+4 =  7+3 =  25 / 5 = | Not numerate at all ………………………………………..1  Adds with difficulty…………………….…………………..2  Adds with ease………………………………………..……3  Division with ease…………………………………………..4 | |

|  |  | No – No difficulty | Yes – some difficulty | Yes – a lot of difficulty | Cannot do at all |
| --- | --- | --- | --- | --- | --- |
| 11 | Do you have difficulty seeing, even if wearing glasses? | 1 | 2 | 3 | 4 |
| 12 | Do you have difficulty hearing? | 1 | 2 | 3 | 4 |
| 13 | Do you have difficulty walking or climbing? | 1 | 2 | 3 | 4 |
| 14 | Do you have difficulty remembering or concentrating? | 1 | 2 | 3 | 4 |
| 15 | Do you have difficulty speaking? | 1 | 2 | 3 | 4 |

| **HOW YOU FEEL**  **Now we have come questions about how you feel. I am going to read three statements and ask you to choose which describes you best for the past two weeks.** | | | |
| --- | --- | --- | --- |
| **Item** | **1** | **2** | **3** |
| 201a | I am never sad | I am sad once in a while. | I am sad many times |
| 201b | Nothing will ever work out for me. | I am not sure if things will work out for me. | Things will work out for me OK. |
| 201c | I do most things OK | I do many things wrong. | I do everything wrong. |
| 201d | I have fun in many things. | I have fun in some things | Nothing is fun at all. |
| 201e | I am important to my family | I am not sure if I am important to my family. | My family is better off without me. |
| 201f | I hate myself | I do not like myself | I like myself |
| 201g | All bad things are my fault | Many bad things are my fault | Bad things are not usually my fault |
| 201h | I do not think about killing myself | I think about killing myself but would not do it. | I want to kill myself. |
| 201i | I feel like crying every day | I feel like crying many days. | I feel like crying once in a while or never. |
| 201j | I feel annoyed all the time | I feel annoyed many times | I am almost never annoyed |
| 201k | I like being with people | I do not like being with people many times | I do not want to be with people at all. |
| 201l | I cannot make up my mind about things | It is hard to make up my mind about things. | I make up my mind about things easily. |
| 201m | I look ok | There are some bad things about my looks | I look ugly |
| 201n | I have to push myself all the time to do my schoolwork | I have to push myself many times to do my schoolwork | Doing schoolwork is not a big problem |
| 201o | I have trouble sleeping every night | I have trouble sleeping many nights | I sleep pretty well |
| 201p | I am tired once in a while | I am tired many days | I am tired all the time |
| 201q | Most days I do not feel like eating | Many days I do not feel like eating | I eat pretty well |
| 201r | I do not worry about aches and pains | I worry about aches and pains many times | I worry about aches and pains all the time |
| 201s | I do not feel alone | I feel alone many times | I feel alone all the time |
| 201t | I never have fun at school | I have fun at school only once in a while | I have fun at school many times |
| 201u | I have plenty of friends | I have some friends but I wish I had more | I do not have any friends |
| 201v | My schoolwork is alright | My schoolwork is not as good as before | I do very badly in subjects I used to be good in |
| 201w | I can never be as good as other kids | I can be as good as other kids if I want to | I am just as good as other kids |
| 201x | Nobody really loves me | I am not sure if anybody loves me | I am sure that somebody loves me |
| 201y | It is easy for me to get along with friends | I get into arguments with friends many times | I get into arguments with friends all the time |
| 201z | I fall asleep during the day all the time | I fall asleep during the day many times | I almost never fall asleep during the day |
| 201aa | Most days I feel like I can’t stop eating | Many days I feel like I can’t stop eating | My eating is OK |
| 201ab | It is easy for me to remember things | It is a little hard to remember things | It is very hard to remember things |

| **FIGHTING AMONG CHILDREN** | | | | | |
| --- | --- | --- | --- | --- | --- |
| **How often within the past month has another child done these things to you?** | | **Never** | **Once** | **2or 3 times** | **4 or more times** |
| 301 | Called me bad names | 0 | 1 | 2 | 3 |
| 302 | Tried to get me into trouble with my friends | 0 | 1 | 2 | 3 |
| 303 | Took something of mine without permission | 0 | 1 | 2 | 3 |
| 304 | Made fun of me because of my appearance | 0 | 1 | 2 | 3 |
| 305 | Made fun of me for some reason apart from my appearance | 0 | 1 | 2 | 3 |
| 306 | Tripped me to make me fall | 0 | 1 | 2 | 3 |
| 307 | Pushed me to hurt me | 0 | 1 | 2 | 3 |
| 308 | Hurt me physically | 0 | 1 | 2 | 3 |
| 309 | Beat me so badly that I was injured | 0 | 1 | 2 | 3 |
| 310 | Deliberately broken something that belongs to me | 0 | 1 | 2 | 3 |
| 311 | Tried to make other children turn against me | 0 | 1 | 2 | 3 |
| 312 | Stole something from me | 0 | 1 | 2 | 3 |
| 313 | Refused to talk to me | 0 | 1 | 2 | 3 |
| 314 | Made other people not talk to me | 0 | 1 | 2 | 3 |
| 315 | Deliberately damaged something of mine | 0 | 1 | 2 | 3 |
| 316 | Swore at me | 0 | 1 | 2 | 4 |
|  | **Where did you experience things in the last 4 weeks?** | **Never** | **Once** | **2or 3 times** | **4 or more times** |
| 317 | In a school classroom | 0 | 1 | 2 | 3 |
| 318 | Outside school | 0 | 1 | 2 | 3 |
| 319 | In the streets | 0 | 1 | 2 | 3 |
| 320 | At home | 0 | 1 | 2 | 3 |

| **PUNISHMENT SCHOOL** | | **Never** | **Once** | **2 or 3 times** | **4 or more times** |
| --- | --- | --- | --- | --- | --- |
| 321 | How often within the past month were you slapped, hit or beaten or otherwise physically punished by a teacher? Was it never, once, 2-3 times or more often? | 0 | 1 | 2 | 3 |
| 322 | How often within the past month did a teacher twist your ear? Was it never, once, 2-3 times or more often? | 0 | 1 | 2 | 3 |
| 323 | How often within the past month did a teacher make you stand on a bench? Was it never, once, 2-3 times or more often? | 0 | 1 | 2 | 3 |
| 324 | How often within the past month did a teacher make you run around as a punishment? Was it never, once, 2-3 times or more often? | 0 | 1 | 2 | 3 |
| 325 | How often within the past month did a teacher make you kneel down in class or outside? Was it never, once, 2-3 times or more often? | 0 | 1 | 2 | 3 |
| 326 | In a past month did a teacher hit you with a stick, whip or other object? Was it never, once, 2-3 times or more often? | 0 | 1 | 2 | 3 |
| **PUNISHMENT AT HOME** | | **Never** | **Once** | **2 or 3 times** | **4 or more times** |
| 327 | How often within the past 4 weeks were you slapped, hit or beaten or otherwise physically punished by a parent? Was it never, once, 2-3 times or more often? | 0 | 1 | 2 | 3 |
| 328 | In the past 4 weeks have you been beaten so hard at home that you were injured? Was it never, once, 2-3 times or more often? | 0 | 1 | 2 | 3 |

| **FIGHTING AND PUNISHMENT IN THE FAMILY** | | **Never** | **Once** | **2 or 3 times** | **4 or more times** |
| --- | --- | --- | --- | --- | --- |
| **329** | **In the last 4 weeks,** how often have you seen or heard that your father had a physical fight with another man? Was it never, once, 2-3 times or more often? | 0 | 1 | 2 | 3 |
| **330** | **In the last 4 weeks,** how often have you seen or heard your father hit your mother? Was it never, once, 2-3 times or more often? | 0 | 1 | 2 | 3 |
| **331** | **In the last 4 weeks,** how often have you seen or heard your mother being beaten by any of the family members? Was it never, once, 2-3 times or more often? | 0 | 1 | 2 | 3 |
| **332** | **In the last 4 weeks,** how often did you go to school without breakfast because of lack of food at home? Was it never, once, 2-3 times or more often? | 0 | 1 | 2 | 3 |
| **333** | **In the last 4 weeks,** how often do you go to sleep without dinner because of lack of food at home? Was it never, once, 2-3 times or more often? | 0 | 1 | 2 | 3 |

| **FIGHTING AMONG CHILDREN** | | | | | | | |
| --- | --- | --- | --- | --- | --- | --- | --- |
| **How often within the past 4 weeks have you** | | | **Never** | | **Once** | **Few Times** 2 or 3 | **Many Times** 4 or more |
| 401 | Called another child bad names | | 0 | | 1 | 2 | 3 |
| 402 | Tried to get another child into trouble with friends | | 0 | | 1 | 2 | 3 |
| 403 | Upset or annoyed another child by taking something of theirs without permission | | 0 | | 1 | 2 | 3 |
| 404 | Made fun of another child because of their appearance | | 0 | | 1 | 2 | 3 |
| 405 | Made fun of another child for some reason apart from their appearance | | 0 | | 1 | 2 | 3 |
| 406 | Tripped another child to make him or her fall | | 0 | | 1 | 2 | 3 |
| 407 | Pushed another child to hurt him or her | | 0 | | 1 | 2 | 3 |
| 408 | Hurt another child physically | | 0 | | 1 | 2 | 3 |
| 409 | Beat another child so badly that they were injured | | 0 | | 1 | 2 | 3 |
| 410 | Deliberately broken something that belong to another child | | 0 | | 1 | 2 | 3 |
| 411 | Tried to make other children turn against another child | | 0 | | 1 | 2 | 3 |
| 412 | Stolen something from another child | | 0 | | 1 | 2 | 3 |
| 413 | Refused to talk to another child | | 0 | | 1 | 2 | 3 |
| 414 | Made other children not talk to another child | | 0 | | 1 | 2 | 3 |
| 415 | Deliberately damaged something of another child’s | | 0 | | 1 | 2 | 3 |
| 416 | Swear at another child | | 0 | | 1 | 2 | 3 |
| **CHILD PUNISHMENT** | | | | | | | |
| We have some statements next. I will read them out and I would like to know for each if you strongly disagree, disagree, agree or strongly agree | | **Strongly Disagree** | | **Disagree** | | **Agree** | **Strongly Agree** |
| 417 | I think that if a child disobeys their parents they should be beaten | 1 | | 2 | | 3 | 4 |
| 418 | I think that if a child gets into fights their parents should beat them | 1 | | 2 | | 3 | 4 |
| 419 | I think that if a child talks back to their parents they should be punished by being beaten | 1 | | 2 | | 3 | 4 |
| 420 | I think a child who misbehaves at school should be beaten | 1 | | 2 | | 3 | 4 |
| 421 | I think that if a child hurts me I should hurt them back | 1 | | 2 | | 3 | 4 |

| **POSITION OF WOMEN** | | | | | |
| --- | --- | --- | --- | --- | --- |
| We have some statements next. I will read them out and I would like to know for each if you strongly disagree, disagree, agree or strongly agree | | **Strongly Disagree** | **Disagree** | **Agree** | **Strongly Agree** |
| 501 | I think girls in my family should go to school | 1 | 2 | 3 | 4 |
| 502 | I think the husbands in my family should give permission to give their wives to go to the clinic | 1 | 2 | 3 | 4 |
| 503 | I think the husbands in my family should listen to their wives’ opinion on schooling | 1 | 2 | 3 | 4 |
| 504 | I think the wives in my family should have a say in how money in the family is spent | 1 | 2 | 3 | 4 |
| 505 | I think the wives in my family should be able to ask a religious scholar about religious issues | 1 | 2 | 3 | 4 |
| 506 | I think the husbands in my family should respect the opinion of their wives on matters related to income generating work | 1 | 2 | 3 | 4 |
| 507 | I think a husband in my family should be kind and caring toward the women in his family | 1 | 2 | 3 | 4 |
| 508 | I think that the wives in my family should always obey their husbands | 1 | 2 | 3 | 4 |
| 509 | I think that if a wife in my family does something wrong her husband has the right to punish her | 1 | 2 | 3 | 4 |

| **Circle the number that describes how much you agree or disagree with each statement.** | | **Strongly Disagree** | **Disagree** | **Agree** | **Strongly Agree** |
| --- | --- | --- | --- | --- | --- |
| 510 | **I think women should be able to participate in**  Weddings | 1 | 2 | 3 | 4 |
| 511 | Neighbourhood events | 1 | 2 | 3 | 4 |
| 512 | Skills training (e.g. computer skills, embroidery) | 1 | 2 | 3 | 4 |
| 513 | Income generating activities | 1 | 2 | 3 | 4 |

|  | Now we have some final questions that we would like you to answer. For each of the statements and we would like to know if you strongly agree, agree, disagree or strongly disagree with these | | | | |
| --- | --- | --- | --- | --- | --- |
|  |  | *Strongly disagree*  1 | *Disagree*  2 | *Agree*  3 | *Strongly agree*  4 |
| 514 | I can think of many ways to get out of a difficult situation | 1 | 2 | 3 | 4 |
| 515 | I put lots of energy into pursing my goals | 1 | 2 | 3 | 4 |
| 516 | There are lots of ways around any problem | 1 | 2 | 3 | 4 |
| 517 | I can think of many ways to get the things in life that are important to me | 1 | 2 | 3 | 4 |
| 518 | Even when others get discouraged, I know I can find a way to solve the problem | 1 | 2 | 3 | 4 |
| 519 | I meet the goals that I set for myself | 1 | 2 | 3 | 4 |

|  |  |  |
| --- | --- | --- |
| 520 | In the last year, how often did you attend the peace education classes at school? Was it: every week, many weeks, occasionally, or not at all | Every week……………..1  Many weeks…………….2  Occasionally…………….3  Not at all…………………4 |
| 521 | In the past year how many peace education classes did you attend? | 1-5…………………….1  6-10…………..………..2  11-20…………………..3  More than 20…………..4 |
| 522 | Did you find the lessons taught in the peace education classes were useful to your life? Would you say they were very useful, somewhat useful, a little useful or not useful at all? | Very useful……………..1  Somewhat useful…..…….2  A little useful …………….3  Not useful at all…………4 |
| 523 | Thinking back to the year before last year, how often did you attend the peace education classes at school? Was it: every week, many weeks, occasionally, or not at all | Every week……………..1  Many weeks…………….2  Occasionally…………….3  Not at all…………………4 |
| 524 | Thinking back to the year before last year, can you recall how many peace education classes you attended? | 1-5…………………….1  6-10…………..………..2  11-20…………………..3  More than 20…………..4 |

THANK YOU FOR HELPING US TO HELP OTHER CHILDREN
